# A Genetic Screen for Dihydropyridine (DHP)-Resistant Worms Reveals New Residues Required for DHP-Blockage of Mammalian Calcium Channels

**DOI:** 10.1371/journal.pgen.1000067

**Published:** 2008-05-09

**Authors:** Trevor C. Y. Kwok, Kwokyin Hui, Wojciech Kostelecki, Nicole Ricker, Guillermo Selman, Zhong-Ping Feng, Peter John Roy

**Affiliations:** 1 Department of Molecular Genetics, The Terrence Donnelly Centre for Cellular and Biomolecular Research, University of Toronto, Toronto, Ontario, Canada; 2 Department of Physiology, University of Toronto, Toronto, Ontario, Canada; The Jackson Laboratory, United States of America

## Abstract

Dihydropyridines (DHPs) are L-type calcium channel (Ca_v_1) blockers prescribed to treat several diseases including hypertension. Ca_v_1 channels normally exist in three states: a resting closed state, an open state that is triggered by membrane depolarization, followed by a non-conducting inactivated state that is triggered by the influx of calcium ions, and a rapid change in voltage. DHP binding is thought to alter the conformation of the channel, possibly by engaging a mechanism similar to voltage dependent inactivation, and locking a calcium ion in the pore, thereby blocking channel conductance. As a Ca_v_1 channel crystal structure is lacking, the current model of DHP action has largely been achieved by investigating the role of candidate Ca_v_1 residues in mediating DHP-sensitivity. To better understand DHP-block and identify additional Ca_v_1 residues important for DHP-sensitivity, we screened 440,000 randomly mutated *Caenorhabditis elegans* genomes for worms resistant to DHP-induced growth defects. We identified 30 missense mutations in the worm Ca_v_1 pore-forming (α_1_) subunit, including eleven in conserved residues known to be necessary for DHP-binding. The remaining polymorphisms are in eight conserved residues not previously associated with DHP-sensitivity. Intriguingly, all of the worm mutants that we analyzed phenotypically exhibited increased channel activity. We also created orthologous mutations in the rat α_1C_ subunit and examined the DHP-block of current through the mutant channels in culture. Six of the seven mutant channels examined either decreased the DHP-sensitivity of the channel and/or exhibited significant residual current at DHP concentrations sufficient to block wild-type channels. Our results further support the idea that DHP-block is intimately associated with voltage dependent inactivation and underscores the utility of *C. elegans* as a screening tool to identify residues important for DHP interaction with mammalian Ca_v_1 channels.

## Author Summary

L-type calcium channels are important drug targets because they regulate many physiological processes throughout the body. For example, L-type calcium channels regulate cardiac myocytes and vascular smooth muscle contraction. Antagonists are therefore commonly used to lower blood pressure and treat other related ailments. Despite their medical importance, the mechanism by which L-type antagonists inactivate calcium channels is not fully understood, due in large part to the lack of a channel crystal structure. Here, we present the first large-scale genetic screen for L-type calcium channel residues that are important for sensitivity to a new drug analog that we discovered called nemadipine. We performed the screen using nematodes, and then recreated similar mutations in a mammalian channel to investigate how the mutant residues alter interactions with the antagonists using electrophysiological techniques. Together, our analyses revealed eight new L-type calcium channel residues that are important for DHP-sensitivity and highlight the utility of using a simple animal model system for understanding how drugs interact with their targets.

## Introduction

**Figure 1:**
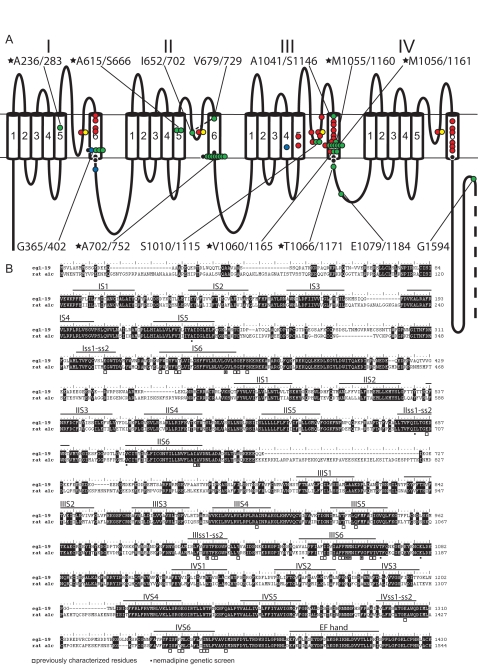
Topology diagram and sequence alignment of the Ca_v_1 calcium channel α_1_ subunit. (A) A topology diagram of the *C. elegans* Ca_v_1 channel α_1_ subunit EGL-19 with residues of interest shown. Residues known to be involved in DHP interaction are in red. Blue circles are the previously characterized hypermorphic residues in EGL-19. Yellow circles are the residues that form the calcium ion filter. Black circles are residues involved in voltage dependent slow inactivation. Green circles are the EGL-19 polymorphisms isolated in our forward genetic screen and labeled with the corresponding residue number (worm residue/rat residue) and a star if analyzed further through phenotypic and physiological analyses. See Tables 1 and 2 for more information about DHP-sensitive residues. (B) Sequence alignment between EGL-19 (GenBank accession number: NM_001027908) and the orthologous rat brain Ca_v_1.2 α_1C_ subunit (GenBank accession number: M67515). The predicted positions of the transmembrane segments (IS1-IVS6), channel pore (SS1–SS2) and the EF hand are shown and are based on the EGL-19 sequence[29]. Identical residues between the two sequences are highlighted in black. An open box beneath the amino acid shows the site of the corresponding coloured circle from the topology diagram in (A). A black box below the amino acid indicates the sites of the mutations we isolated in EGL-19. The alignment is shown only slightly past the EF hand as sequence conservation for the C-terminal tail is low after this point.

Calcium influx into the cell can make both an immediate and a long-term impact on many cellular processes, including membrane excitability, neurotransmitter release, muscle contraction, transcription, and proliferation [1]. It is therefore not surprising that calcium current through calcium-permeable channels is tightly regulated. Calcium channels of the Ca_v_ family, for example, regulate current through voltage dependent mechanisms and exist in three major biophysical states: a resting (non-conducting) state, an activated (conducting) state that is triggered by a rapid change of voltage, and an inactivated (non-conducting) state that follows the conducting state but is refractory to activation. Ca_v_ channels are composed of multiple subunits that regulate calcium current through the pore-forming and voltage-sensing α_1_ subunit, which has four repeating domains (called I–IV), each with six transmembrane segments designated S1–S6 [2],[3] (Figure 1). The most widely-expressed Ca_v_ channels are the L-type, otherwise known as Ca_v_1 channels. In humans, alterations in Ca_v_1 abundance or function result in a variety of diseases, including hypertension [4], Timothy Syndrome [5], and congenital stationary night blindness [6]. A better understanding of Ca_v_1 function and how this function can be modulated by drug-like molecules may therefore lead to better treatments of Ca_v_1-related diseases.

A distinguishing feature of Ca_v_1 channels is their sensitivity to 1,4-dihydropyridines (DHPs), which bind to the α_1_ subunit and are commonly prescribed to reduce hypertension in humans [7]. Exactly how DHPs physically interact with the α_1_ subunit is incompletely understood, in large part because the crystal structure of any Ca_v_1 α_1_ subunit remains elusive. However, Ca_v_1 residues that facilitate DHP interaction have been identified through the analyses of candidate domains and residues. For example, swapping domains IIIS5, IIIS6, and IVS5-IVS6 from an L-type to a non-L-type channel is sufficient to confer DHP-sensitivity to otherwise insensitive Ca_v_ channels [8],[9],[10]. Individual Ca_v_1 residues present in DHP-sensitive, but not insensitive channels, have also been extensively investigated to determine if they are necessary and sufficient for DHP-sensitivity [11],[12],[13],[14]. In this way, four residues in domain IS6, two residues in IIIS5, eight residues in IIIS6, and six residues in IVS6 were shown to be important for DHP binding to the α_1_ subunit of the Ca_v_1 channel in culture [11],[14],[15],[16],[17],[18] (summarized in Table 1).

**Table 1:** A compendium of Ca_v_1.2 residues that may impact DHP interactions *in vivo*.

Effect	Worm Residue^a^	Rat Residue^b^	Region	Identified in our Screen?	Reference
**Hypermorphic Channel**					
	G365	G402	IS6	Y	Lee et al., 1997
	S372	S409	I–II linker	-	Lee et al., 1997
	A906	A1011	IIIS4	-	Lee et al., 1997
**Required for Slow Inactivation**					
	G365	G402	IS6	Y	Splawski et al., 2005
	S368	S405	IS6	-	Shi & Soldatov, 2002
	G369	G406	IS6	-	Splawski et al., 2004
	I701	I751	IIS6	-	Hohaus et al., 2005
	A702	A752	IIS6	Y	Soldatov et al., 2000
	V1063	V1168	IIIS6	-	Shi & Soldatov, 2002
	I1064	I1169	IIIS6	-	Shi & Soldatov, 2002
	I1364	I1478	IVS6	-	Shi & Soldatov, 2002
**Required for Allosteric Enhancement of DHP Interaction by Calcium Occupancy**					
	E326	E363	ISS1–SS2	-	Peterson & Catterall, 2006
	E656	E706	IISS1–SS2	-	Peterson & Catterall, 2006
	F1007	F1112	IIISS1–SS2	-	Peterson & Catterall, 2006
	S1010	S1115	IIISS1–SS2	Y	Yamagushi et al., 2000, 2003
	F1012	F1117	IIISS1–SS2	-	Peterson & Catterall, 2006
	E1013	E1118	IIISS1–SS2	-	Peterson & Catterall, 1995
	Y1020	Y1125	IIISS1–SS2	-	Peterson & Catterall, 2006
	E1302	E1419	IVSS1–SS2	-	Peterson and Catterall, 1995
**Required for DHP Interaction**					
	P344	P381	IS6	-	Lacinova et al., 1999
	I346	V383	IS6	-	Lacinova et al., 1999
	V349	V386	IS6	-	Lacinova et al., 1999
	T350	S387	IS6	-	Lacinova et al., 1999
	T934	T938	IIIS5	-	Mitterdorfer et al., 1996
	Q1039	Q1043	IIIS5	-	Wappl et al., 2001
	F1047	Y1152	IIIS6	-	Hockerman et al., 1997
	I1048	I1153	IIIS6	-	Hockerman et al., 1997
	I1051	I1156	IIIS6	-	Hockerman et al., 1997
	F1053	F1158	IIIS6	-	Hockerman et al., 1997
	F1054	F1159	IIIS6	-	Hockerman et al., 1997
	M1055	M1160	IIIS6	Y	Hockerman et al., 1997
	M1056	M1161	IIIS6	Y	Hockerman et al., 1997
	V1060	V1165	IIIS6	Y	Wappl et al., 2001
	I1346	I1460	IVS6	-	Peterson et al., 1996
	F1349	Y1463	IVS6	-	Hockerman et al., 1997
	M1350	M1464	IVS6	-	Hockerman et al., 1997
	V1356	I1470	IVS6	-	Hockerman et al., 1997
	I1357	I1471	IVS6	-	Hockerman et al., 1997
	N1358	N1472	IVS6	-	Hockerman et al., 1997

a The numbers are based on the worm Ca_v_1 channel (EGL-19) protein sequence (GenBank NM_001027908).

b The numbers are based on the rat brain Ca_v_1.2 α_1C_ subunit protein sequence (GenBank M67515).

DHPs are thought to block calcium current by inducing allosteric structural changes in the channel that engages the channel in the inactivated state [18],[19] and promotes high affinity interaction with a single calcium ion within the pore [20],[21],[22], thereby preventing further ion flow. Calcium ions are bound by the channel through four negatively charged glutamate residues (called the calcium selectivity filter), which reside within four membrane-embedded intersegmental loops between S5 and S6 (called SS1–SS2) of each domain (Table 1). Together, the four SS1–SS2 domains form the extracellular-facing ‘outer pore’ of the channel (see Figure 1). The relationship between the DHP and the selectivity filter is bidirectional: DHP-binding facilitates a high affinity interaction between the selectivity filter and a calcium ion, and the coupling of the selectivity filter to calcium promotes a high-affinity interaction between the DHP and the channel [21],[23],[24]. A candidate analysis of residues surrounding the glutamate selectivity filter revealed several residues that are also important for high-affinity DHP interaction with the channel in culture [21],[24],[25],[26] (Table 1).

Clearly, the candidate approach has been fruitful in identifying residues important for DHP antagonism of Ca_v_1 channels. However, as the exact molecular mechanism of DHP block remains incompletely understood, the search for additional residues involved in DHP-sensitivity might add to our understanding of DHP-Ca_v_1 interaction. We therefore screened random mutants of the tiny nematode worm *C. elegans* for those animals that are resistant to the effects of a novel DHP analog called nemadipine in the hopes of identifying new Ca_v_1 residues important for DHP-sensitivity.

We recently discovered nemadipine through a screen for new biologically active small molecules [27],[28]. Through a variety of genetic approaches, we demonstrated that the major target of nemadipine is EGL-19 [27], which is the sole Ca_v_1 channel α_1_ subunit encoded by the *C. elegans* genome [29]. We also found that nemadipine can antagonize vertebrate Ca_v_1 channels with a similar potency as an FDA-approved DHP in chick ciliary neurons [27]. However, none of the FDA-approved Ca_v_1 antagonists examined, including nifedipine, nicardipine, felodipine, and nimodipine, elicit robust phenotypes in whole worms. Worm insensitivity to these DHPs is not likely due to Ca_v_1 divergence since nifedipine can antagonize EGL-19 in dissected worms [29]. Instead, we discovered that most FDA-approved DHPs fail to accumulate in whole worms [27], consistent with the idea that *C. elegans* has extensive xenobiotic defenses. Thus, nemadipine is a unique reagent to genetically probe interactions between DHPs and Ca_v_1 channels *in vivo*.

Here, we present our genetic screen of 440,000 mutant *C. elegans* genomes for animals that are resistant to nemadipine. Strikingly, 30 of the 35 mutants that we characterized have missense polymorphisms in the *egl-19* gene. Only one of these mutations is in a non-conserved residue. Eleven of the mutations correspond to residues of the Ca_v_1 channel that are known to be required for DHP interaction with mammalian channels. The remaining mutations are in nine residues not previously associated with DHP interaction, eight of which are conserved. Behavioral and developmental analyses of 12 of our worm mutants revealed that all exhibit phenotypes consistent with increased channel activity. For seven of these worm mutations, we created orthologous mutations in the rat brain Ca_v_1.2 channel and examined the ability of DHPs to block ion current through these mutant channels using whole-cell voltage clamp analysis in culture. Two of the mutant channels have decreased sensitivity to the DHP block, and six disrupt the ability of the DHP to block ion current completely. Thus, screening for worm mutants that are resistant to nemadipine is a viable and novel approach to identify mammalian Ca_v_1 residues that are important for DHP sensitivity.

## Results

### A Forward Genetic Screen for DHP-Resistant *C. elegans* Mutants Yields 30 Polymorphisms in EGL-19

Previous work demonstrated the utility of using *C. elegans* calcium channels as a model to better understand mammalian channel function [30]. We therefore performed a forward genetic screen for *C. elegans* mutants that are resistant to nemadipine-induced growth retardation to identify residues in the Ca_v_1 calcium channel required for DHP sensitivity. A total of 440,000 haploid mutagenized wild type genomes were screened and 55 candidate mutants were isolated. Homozygous lines were established for 35 of these mutants and polymorphisms were identified in *egl-19* for 30 (Figure 1 and Table 2). Four of the remaining mutants are genetically linked to *egl-19* on chromosome IV, but no polymorphism in *egl-19* could be found (data not shown). The remaining mutant is not linked to *egl-19* and is not discussed further here. Upon out-crossing select mutants, we found that all of them exhibited increased resistance to nemadipine in two separate dose-response assays (Table 2, Figure S1), confirming that they are indeed DHP-resistant mutants.

**Table 2:** A list of previously known and newly identified *egl-19* alleles.

allele	worm mutation^a^	rat residue^a^	region mutated	DHP-associated?^b^	Egl IC_50_ c	Vab IC_10_ c
*-*	wild-type	-	-	-	1.2	2.1
* *ad695*	A906V	A1011	IIIS4	Y [27]	50	32
* *n2368*	G365R	G402	IS6	Y [27]	>>50	>>50
* *ad1006*	-	-	-	-	<<0 uM	0.2 uM
* *n582*	A899H	R1004	IIIS4	-	<<0 uM	0.2 uM
* *tr81*	A236V	*A283	IS5	N	9	39
*tr134*	G365R	G402	IS6	Y [27]	-	-
*tr135*	G365R	G402	IS6	-	-	-
*tr136*	G365R	G402	IS6	-	-	-
* *tr73*	A615V	*S666	IIS5	N	∼50	>>50
* *tr93*	A615V	S666	IIS5	-	∼50	>>50
*tr72*	I652F, V679I	I702, V729	IISS1–SS2, IIS6	N/N	-	-
* *tr79*	A702T	*A752	IIS6	N	>>50	>>50
*tr84*	A702T	A752	IIS6	-	-	-
* *tr132*	A702T	A752	IIS6	-	>>50	>>50
*tr85*	A702V	A752	IIS6	-	-	-
* *tr76*	A702V	*A752	IIS6	-	>>50	>>50
* *tr86*	A702V	A752	IIS6	-	>>50	>>50
*tr87*	A702V	A752	IIS6	-	-	-
*tr88*	A702V	A752	IIS6	-	-	-
*tr70*	S1010L	S1115	IIISS1–SS2	Y [25]	-	-
*tr75*	S1010L	S1115	IIISS1–SS2	-	-	-
*tr77*	A1041V	S1146	IIIS6	N	-	-
* *tr92*	M1055L	*M1160	IIIS6	Y [14]	>50	>>50
* *tr69*	M1056I	*M1161	IIIS6	Y [14]	>50	>>50
* *tr89*	M1056I	M1161	IIIS6	-	∼50	>>50
*tr90*	M1056I	M1161	IIIS6	-	-	-
*tr137*	M1056I	M1161	IIIS6	-	-	-
*tr138*	M1056I	M1161	IIIS6	-	-	-
*tr91*	M1056I	M1161	IIIS6	-	-	-
* *tr71*	V1060L	*V1165	IIIS6	Y [18]	3	>>50
*tr78*	V1060G	V1165	IIIS6	-	-	-
* *tr74*	T1066I	*T1171	c-term IIIS6	N	7.75	>50
*tr139*	E1079K	E1184	c-term IIIS6	N	-	-
*tr140*	G1594D	n.a.	c-term tail	N	-	-

a Residues numbered as in Table 1.

b If the residue was previously implicated in DHP-sensitivity, a ‘Y’ (yes) is displayed along with the reference.

c The Egl IC_50_ and the Vab IC_10_ are determined from nemadipine dose response assays measuring the number of egg laying defective (Egl) animals and animals with variable abnormal morphology (Vab) as described in Materials and Methods.

*: These are the alleles that are further characterized through phenotypic and physiological analyses.

The DHP binding site on the Ca_v_1 channel pore-forming α_1c_-subunit of vertebrate channels is defined by at least nine residues that are both necessary and sufficient to confer DHP-sensitivity to a non-L-type calcium channel (Figure 1, Table 1). Two of these residues reside in helix S5 of domain III (called IIIS5), three reside in IIIS6 and four in IVS6 [12],[13],[14]. Other studies have shown the involvement of additional residues required for DHP interactions (Table 1) [15],[18]. From our screen for nemadipine-resistant mutants, we found ten mutants with polymorphisms within the IIIS6 domain of EGL-19. Six of these mutate a single EGL-19 residue, M1056 (rat α_1c_ residue M1161), critical for high-affinity DHP binding to the vertebrate channel [11],[14]. We found a seventh mutation in EGL-19 residue M1055 (rat α_1c_ residue M1160) that is also necessary for DHP binding to the vertebrate channel [11]. An eighth polymorphism was detected in the EGL-19 residue A1041 (rat α_1c_ residue S1146). Previous work demonstrated that a rat α_1c_ S1146A mutation does not disrupt DHP binding [25], which is perhaps not surprising given that the alanine is conserved in some Ca_v_1 channels [25]. However, no one has yet tested if other residues in position 1146, such as the valine substitution found in our mutant, would disrupt interactions with DHPs. Finally, two polymorphisms were identified in the EGL-19 residue V1060 (rat α_1c_ residue V1165) within IIIS6 that has yet to be tested for its role in DHP block. Our finding that seven of the ten mutations that we identified within the IIIS6 region are required for DHP-interaction in mammalian Ca_v_1.2 channels validates our genetic approach for identifying residues required for DHP-sensitivity, and suggests that the new residues we uncovered are also important determinants of DHP sensitivity.

High-affinity DHP binding is coupled to the interaction between the selectivity filter in the outer pore of the α_1_ subunit and calcium ions (see introduction). Mutating the glutamate residues and several of the surrounding residues of the selectivity filter not only dramatically reduces the binding of calcium ions, but disrupts DHPs calcium-dependent affinity for the channel [21]. For example, the S1115 residue within SS1-SS2 of the rat α_1c_ channel that precedes the key glutamate in domain III has been shown to be necessary for DHP-sensitivity (Figure 1, Table 1) [25],[26]. We found that two of the nemadipine-resistant mutants are polymorphic within the EGL-19 residue S1010 that is equivalent to the rat α_1c_ residue S1115 (Figure 1; Table 2). We also isolated a mutant with a polymorphism in a similar position in domain II (EGL-19 residue I652; rat α_1c_ residue I702). However, this mutant had a second polymorphism in V679 (rat residue V729), confounding the attribution of any mutant property to either polymorphism. Nevertheless, these results suggest that disrupting residues within the outer pore can reduce DHP-sensitivity *in vivo*.

DHP antagonists have high affinity for Ca_v_1 channels in the inactive state [31]. After voltage dependent opening of the channel, the channel is coordinately inactivated by both calcium-dependent and voltage dependent mechanisms. Previous work has shown that voltage dependent slow inactivation of Ca_v_1.2 channels is mediated by hydrophobic residues in the cytoplasmic end of the S6 transmembrane helices of domains I-IV [32],[33],[34]. Together, these residues are thought to form a hydrophobic voltage-sensitive annulus, the disruption of which leads to prolonged activation of the channel. We found 8 nemadipine-resistant mutants with a polymorphism in EGL-19 A702 within IIS6, which is orthologous to the residue in vertebrates (rat α_1c_ residue A752) that is most important in slow inactivation [32]. We also found two mutants with a polymorphism in the hydrophobic residue V1060 in the IIIS3 domain of EGL-19, which precedes an orthologous residue that contributes to slow inactivation by three positions (rat α_1c_ residue V1168) [33] (Figure 1, Table 1). We also found three mutants with a polymorphism in the hydrophobic residue G365 (rat α_1c_ residue G402) of domain IS6 that is also required for voltage dependent slow inactivation [5],[35]. The same G365R mutation that we isolated was found independently by Michael Hengartner and shown to prolong the activation phase of the mutant EGL-19 channel by Raymond Lee and colleagues *in vivo* [29]. Importantly, the isolation of at least eleven mutants within the voltage-sensitive annulus suggests that DHP-blockage is linked to voltage dependent slow inactivation *in vivo*.

Finally, we identified seven other mutants that have polymorphisms in six residues within regions of the Ca_v_1 channel that have little functional annotation (A236, A615, V679, T1066, E1079, and G1594) (Figure 1, Table 2). Of note, two of these mutant residues are in analogous positions in IS5 and IIS5 (A236 and A615, respectively). Five of the six mutant residues are in highly conserved regions of the channel. The sixth mutant residue (G1594D) is in the carboxy-terminal tail, whose sequence is evolutionarily divergent (Figure 1). We have further investigated three of these mutants through both phenotypic and electrophysiological analysis as described below.

### DHP-Resistant Channels Have Increased Activity *in vivo*

**Figure 2:**
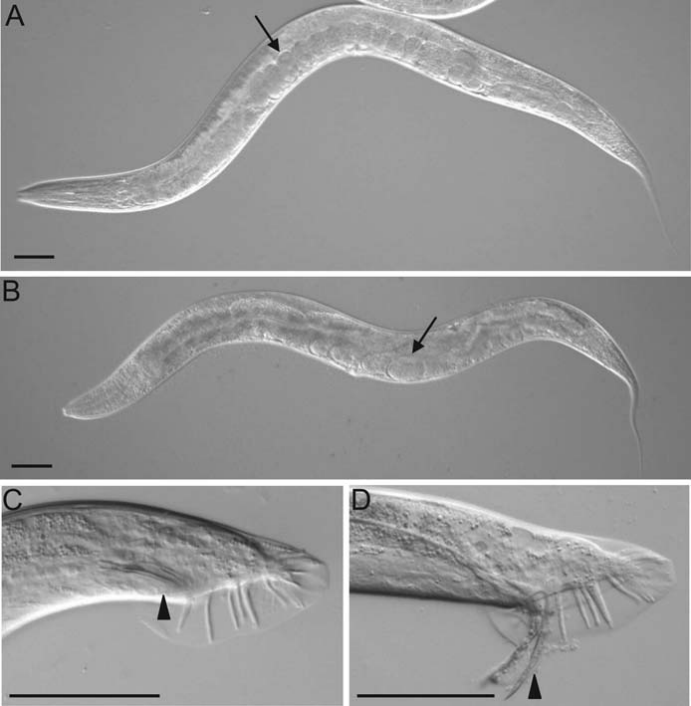
Comparisons of the phenotypes analyzed in *C. elegans*. (A) An N2 adult showing wild-type length and number of embryos (arrow). (B) An *egl-19(tr89)* adult is approximately wild-type in length and contains fewer embryos (arrow). (C) A *him-5(e1490)* adult male showing wild-type tail morphology with the spicules in the normal retracted position (arrowhead). (D) An *egl-19(tr89)* adult male often has spicules that are constantly protracted (arrowhead). All pictures are of animals raised on 0.33% DMSO as L4s. Scale bars, 50 µm.

**Figure 3:**
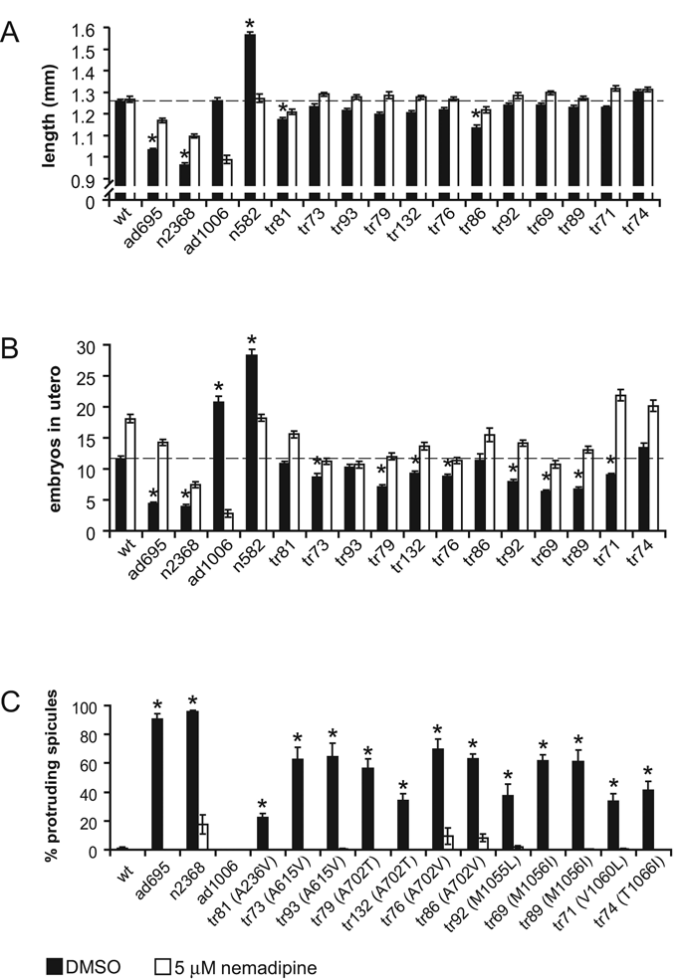
Phenotypic analysis in *C. elegans* shows that the nemadipine-resistant *egl-19* mutations are hypermorphic. A comparison of phenotypes in wild type animals (wt), hypermorphs (*ad695* and *n2368*), the hypomorph *ad1006*, and the nemadipine-resistant mutants isolated in our screen. (A) The length of mutant worms. (B) The number of embryos *in utero* of mutant worms. (C) The protruding spicule phenotype of *egl-19* mutants. The worm polymorphic residue follows the allele designation in brackets. The dotted lines in (A) and (B) represent the phenotype of wild-type worms on DMSO. The sickness of *egl-19* hypomorphs is exacerbated in the presence of nemadipine, accounting for the trend that is opposite than expected in the presence of nemadipine in (A) and (B). Asterisks, *p*<0.001 compared to the negative control in DMSO in (A) and (B), and *p*<0.05 for (C). Error bars, ±s.e.m.

In contrast to traditional electrophysiological approaches that rely on transgenics to investigate DHP interactions, our genomic mutations provided us with a unique opportunity to investigate the consequences of these polymorphisms on channel activity within the context of a whole animal. We reasoned that phenotypic analysis could be used to approximate the level of Ca_v_1 mutant channel activity *in vivo* because the level of Ca_v_1 EGL-19 channel activity has dramatic consequences on the development and behavior of the animals [27],[29]. For example, reduction-of-function mutations or hypomorphs, such as *egl-19(n582)*, are long and flaccid. By contrast, mutant channels that have a prolonged activation phase in electrophysiological studies, such as the *egl-19(n2368)* hypermorphic mutant, are myotonic and consequently shorter than wild type (Figures 2 and 3) [29]. The defects in body size and rigidity in *egl-19* mutants are likely due to altered EGL-19 function in the 95 striated muscles that line the body wall of the worm. The more active the channel, the more pronounced the body size phenotypes become [27],[29]. Thus, if the nemadipine-resistant mutants have unaltered behaviors compared to the wild-type, we might infer that these residues might directly alter DHP binding without altering channel activity. Alternatively, if the nemadipine-resistant mutants behaved more like hypermorphs, then the mutant residues might prolong the activation phase of the channel and confer DHP-resistance indirectly. Hypomorphic mutations were neither expected nor observed, since even weak *egl-19* reduction-of-function mutations dramatically decrease the viability of animals on nemadipine and would not be recovered in our screen [27].

We began our phenotypic analysis by examining the body length of twelve of our mutant strains (Table 2, Figure 3). We chose these 12 mutants because some represent residues that are known to be required for DHP interaction in other systems (worm alleles *tr92*, *tr69*, *tr89*, and *tr71*), while the remaining ones represent potentially new residues not previously implicated in DHP-sensitivity. We measured the length of 20 young adults for each strain and compared their lengths to wild type worms and previously isolated *egl-19* hypermorphs and hypomorphs. None of our twelve mutants were as short as either the weak (*ad695*) or the strong hypermorph (*n2368*) (Figure 3). However, two of our mutants were significantly shorter than wild type animals (*p*<0.001), suggesting that they might be hypermorphic.

Next, we examined behaviors that have a greater dynamic range between wild type animals and previously characterized *egl-19* mutants, as these may be more sensitive to changes in EGL-19 activity. For example, *egl-19* hypomorphs accumulate two-fold or more embryos than the wild-type. Conversely, *egl-19* hypermorphs retain approximately two-fold fewer embryos than the wild-type control (Figures 2 and 3). *egl-19* plays important roles in several cells of the egg-laying circuit, including the eight vulval muscle cells that directly control vulva opening during embryo deposition [27],[29],[36]. Two thirds of our nemadipine-resistant mutants behaved similar to the *egl-19* hypermorphs, retaining significantly fewer embryos than the wild-type (*p*<0.001), consistent with the idea that these mutants are also hypermorphic (Figure 3). Although all of our 12 *egl-19* alleles that we examined exhibit the same trends in the length and egg-retention assays, it is unclear to us why *tr81* and *tr86* behave slightly differently. It may be that background mutations have a mild influence on these two behaviors in these two mutants. As expected, we found a positive correlation (0.64) between the strength of the egg-laying phenotype and the degree of resistance to nemadipine (Table S1).

Finally, we examined how our mutants affect the contraction of a specialized set of muscles in the male tail that regulate spicule protrusion. The spicules are a pair of sclerotized cuticle structures that are housed in the male tail and used during the transfer of sperm to hermaphrodites [37] (Figure 2). The protrusion of each spicule is controlled by a pair of protraction and retraction muscles. *egl-19* is a key regulator of spicule-associated muscles and functions autonomously to regulate their contraction [38]. Nearly all males of a previously characterized *egl-19* hypermorph have protruded spicules, called a Prc phenotype, while *egl-19* hypomorphic males cannot protrude their spicules (Figure 2 and 3) [29],[38]. We found that all of our nemadipine-resistant mutants were obviously Prc (Figure 3). The Prc phenotype of all *egl-19* alleles examined was suppressed by nemadipine (Figure 3). Nemadipine similarly suppressed the shortened length and constitutive egg-laying phenotypes of our *egl-19* mutants (Figure 3). This demonstrates that the observed spicule protrusion, constitutive egg-laying and shortened length phenotypes are all mediated by increased *egl-19* activity. It is noteworthy that the *egl-19* mutations that correspond to the rat α_1c_ residues M1160, M1161, and V1165, which are necessary for high-affinity DHP binding [12],[13],[14], also show increased EGL-19 channel activity. Together, our phenotypic analyses suggest that our nemadipine-resistant mutants have increased channel activity *in vivo*.

### EGL-19 Polymorphic Residues Are also Required for Complete Current Block of Mammalian Ca_v_1.2 Channels by DHPs

We tested if the mutant residues of EGL-19, which confers DHP-resistance and increased channel activity in worms, also alter DHP-sensitivity of a mammalian Ca_v_1.2 channel. We created orthologous mutations in the α_1C_ subunit of the rat Ca_v_1.2 channel and transiently expressed these in tsA201 cells along with auxiliary Ca_v_1.2 channel components β_2a_ and α_2_-δ [39],[40]. Ion currents through both wild-type and mutant channels were then measured in the presence of increasing concentrations of nifedipine, a popular DHP, using whole-cell voltage-clamp electrophysiological techniques (see methods for details). In this way, we could determine if the orthologous rat mutations affect the ability of DHPs to block Ca_v_1.2 channel-mediated ionic current.

**Figure 4:**
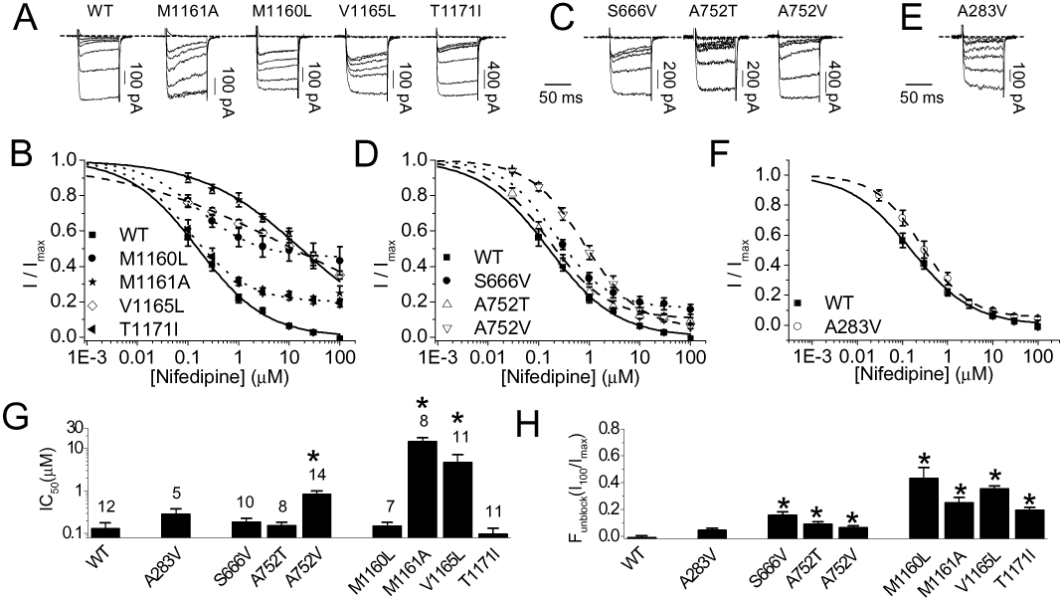
Concentration-dependent block effect of nifedipine on the rat Ca_V_1.2 α_1C_ mutant L-type channels (co-expressed with β_2a_ and α_2_δ). Representatives of barium current records in various nifedipine concentrations (A, C, E) and the mean concentration-effect curves (B, D, F). A & B: the wildtype (WT) and domain III mutants; C. & D: Domain II mutants; and E & F: domain I mutant. The nifedipine concentrations ranged 0, 0.1, 1, 10, 30, 100 µM. The current was elicited by an 80 ms +10 mV depolarizing pulse from a holding potential of −100 mV. In all traces, the baseline was determined by block by 10 µM cadmium (trace with ∼0 pA current). The nifedipine effect was normalized to the unblocked current (I_max_) obtained in the absence of the drug. The concentration-effect curves were fit to a modified Hill equation taking into account the possibility of incomplete current block, as described in the methods. The mean concentration-effect curve for the M1161A mutant, known to be important for DHP block[11],[14], serves as a positive control. Summaries of the IC_50_ values (G), and the residual unblocked current measured at 100 µM nifedipine (H), obtained from the same data presented in Figure B, D, and F. The fraction of current remaining at 100 µM nifedipine (F_unblock_ (I_100_/I_max_)) was used to estimate the completeness of drug block. All the data are presented as mean±s.e.m.; numbers in the parentheses in G reflects the number of independent experiments. * indicates statistical significance relative to the wildtype (p<0.05, one-way ANOVA, *post-hoc* Holm-Sidak).

We first examined polymorphisms in domain IIIS6 residues previously shown to be required for DHP binding. As a positive control, we created a rat α_1C_ M1161A mutation that is known to disrupt the high-affinity interaction between DHPs and the Ca_v_1.2 channel [11],[14]. The M1161A mutation increased the IC_50_ of nifedipine block ∼100-fold compared to the wild type channel, confirming the importance of this residue in DHP sensitivity (Figure 4). The nearby V1165L mutation also increases the IC_50_ of nifedipine block by about 30-fold, consistent with its role in high-affinity DHP interactions [18]. Curiously, a leucine substitution in residue M1160, which is also required for DHP interaction [11], did not increase the IC_50_ of nifedipine block (Figure 4). T1171I, which is cytoplasmic and follows the IIIS6 transmembrane domain, also did not exhibit a shift in the IC_50_. However, both M1160L and T1171I exhibited large residual currents at 100 µM nifedipine, which is sufficient to completely block the ion current through wild type channels (Figure 4). The validity of the residual current was further supported by a comparison to the complete block of current through the respective wild-type and mutant channels by 10 µM cadmium, which is a non-selective pore blocker commonly used to block all current through Ca_v_1 channels [41].

Next, we examined two mutant residues positioned analogously in IS5 (A283V), and IIS5 (A/S666V). Although no significant shift was observed in the IC_50_ response to nifedipine for either channel, the S666V mutant left a significant residual current at saturating concentrations of nifedipine. It is currently unclear why the A283V polymorphic channel behaves similar to the wild-type channel in our assay. Finally, we examined a pair of mutations in a single residue in IIS6 (A752T/V) that contributes to the hydrophobic annulus required for voltage dependent slow inactivation [32]. Consistent with these previous observations, we found that both A752T and A752V yield a significant residual current of about 6-8%, likely reflecting the importance of this site for slow inactivation. Surprisingly, we also found that the IC_50_ of nifedipine block for A752V, but not A752T, increases about five-fold (Figure 4). As expected, we found positive correlations between the IC50 values of the mutant rat channels on nifedipine and the degree of resistance by the respective worm mutants on nemadipine (0.83), and between the amount of residual current of the mutant rat channels and the degree of resistance of the worm mutants (0.63) (Table S1).

In total, two of the seven mutant channels exhibit decreased DHP-sensitivity as indicated by an increased IC_50_ for the DHP-block, and six of the seven mutant channels resist a completely blocked state. These electrophysiology results therefore demonstrate that the identification of Ca_v_1.2 residues that are required for DHP-sensitivity in the worm can reveal residues that are also important for mammalian DHP-sensitivity.

## Discussion

Here, we set out to identify Ca_v_1 residues that are important for DHP sensitivity *in vivo* through a forward genetic screen. Our approach was made possible through our discovery of nemadipine, which is the only DHP known to robustly antagonize Ca_v_1 channels within the context of undissected *C. elegans* worms [27]. By screening for mutants that are resistant to the effects of nemadipine, we identified 30 independent Ca_v_1 polymorphisms that reduce DHP-sensitivity. Upon further investigating 12 of these, we found that all likely increase channel activity in whole worms. Further investigation of how these mutant residues alter the response of a mammalian ortholog to DHP-treatment not only led us to new residues required for DHP sensitivity, but provides *in vivo* support for a previously established idea that the DHP block likely requires voltage dependent inactivation of the channel (see below).

Evidence suggests that at least five of the 14 Ca_v_1 polymorphic residues that we identified are required for physical interactions with DHPs. Four of the residues we identified (corresponding to rat α_1c_ residues S1115, M1160, M1161, and V1165) were previously shown to decrease DHP affinity for the channel when mutated [14],[18],[25]. Additionally, two mutant residues (corresponding to rat α_1c_ residues A752V and V1165L) showed a significant increase in the IC_50_ response to nifedipine in our electrophysiology assay. The observed shift in the IC_50_ is consistent with a decrease in the affinity of the DHP for the mutant channels.

Previous biochemical analyses identified residues within IS6 [15], IIIS5, IIIS6, and IVS6 [11],[16] that are important for DHP binding. However, our genetic screen did not reveal polymorphisms in many of these residues. One reason for this is that our screen was not done to saturation; only six of the 14 residues identified are represented by multiple alleles. It is therefore likely that several residues required for DHP sensitivity *in vivo* remain unidentified, which is proven by the absence of the previously identified A906V nemadipine-resistant mutation [27],[29] from our screen. Second, many of the residues not identified in the genetic screen play only ancillary or weak roles in DHP binding, including those of IS6 [15] and IVS6 [11] and may not confer DHP-resistance by themselves *in vivo*. Third, polymorphisms in each of several IVS6 residues involved in DHP-binding significantly disrupt ion flux through the channel [11] and these types of loss-of-function mutations would not be recovered in our forward genetic screen. Fourth, nemadipine may interact with EGL-19 in slightly different ways compared to how canonical DHPs interact with mammalian channels. If true, this difference might prevent the recovery of mutations of some residues required for high affinity DHP interactions with mammalian channels in our worm screen. However, it remains possible that many of the Ca_v_1.2 residues shown to be required for high-affinity DHP interactions in culture play little or no role in DHP interactions *in vivo*. Regardless, our finding that polymorphisms in S1115, M1160, M1161 and V1165 do confer DHP-resistance *in vivo* adds further weight to existing biochemical and electrophysiological evidence that these residues are critical for DHP interactions.

Several lines of evidence from our work support the previously established idea that DHP-sensitivity of the channel is intimately associated with voltage dependent inactivation [18],[19]. First, our behavioral analyses of mutant worms reveals that polymorphisms within or near the predicted hydrophobic annulus (corresponding to rat α_1C_ polymorphisms G402R, A752T, A752V, V1165L, and T1171I), which are normally required to block the channel through voltage dependent slow inactivation, decrease DHP-sensitivity and increase channel activity *in vivo*. Second, electrophysiological analysis on four of these polymorphisms (A752T, A752V, V1165L, and T1171I) reveal significant residual current in the presence of DHP concentrations that is sufficient to block the wild-type channel in culture and two of these polymorphisms (A752T and T1171I) do not show any change in the IC_50_ for the DHP-block. This suggests that these residues are required to engage the DHP-block without being involved in DHP-sensitivity. Because the A752T mutation is known to disrupt voltage-gated slow inactivation [32] (and T1171 is very close to V1168 and I1169 that are also required for voltage-gated slow inactivation [33]), our observations provide additional support for the previously established idea that the DHP block may be mediated through the same mechanism employed by voltage-gated slow inactivation [18],[19]. Together, our work demonstrates that a forward genetic screen for worms resistant to DHPs is a viable approach for the discovery of residues that are important for mammalian DHP-sensitivity.

## Materials and Methods

### Screening, Mapping, and Phenotypic Analysis of Nemadipine-Resistant Mutants

Candidate mutants were isolated and mapped through their resistance to nemadipine-induced population growth defect as described previously [27],[28]. For this work, we used only the nemadipine-A analog, simply referred to as nemadipine here. To determine the IC_50_ and IC_10_ of the mutants with respect to the egg-laying-retention (Egl) phenotype and the variable abnormal (Vab) morphological defects induced by the compound, a population enriched for adults for each mutant strain was chunked onto MYOB plates containing either nemadipine or only the DMSO solvent as the negative control [28]. Approximately 24 hours later, young adults were picked on to a new nemadipine or DMSO plates. For the Egl assay, adults were scored as either being Egl or non-Egl approximately 24 hours later. For the Vab assay, the L1 and L2 larvae laid by the adults were scored for morphological defects approximately 24 hours later.

To measure length and count embryos *in utero*, roughly 100 L4s of each strain were picked onto plates containing either DMSO or 5 µM nemadipine. Approximately 24 hours later, resulting young adults were photographed at 20× using a Leica MZFLIII microscope with a Retiga 1300 digital camera (Q Imaging). The lengths were measured using Openlab software (Improvision, Inc.). Embryos *in utero* were counted approximately 24 hours after the L4s were place on the plates using a Leica MZFLIII microscope at approximately 80× magnification. For the protruding spicule counts, *him-5(e1490)* doubles were created for each strain. L4 males were picked and put onto either DMSO or 5 µM plates. Approximately 24 hours later, the males were scored as either having protruding spicules (Prc) or non-Prc.

### Creating the Homologous Mutation in the Rat Brain Ca_v_1.2 Channel

The rat brain L-type α_1C_ subunit in pMT2 and β_2a_ and α_2_δ were generous gifts of Dr. Terry Snutch (University of British Columbia, Vancouver, Canada). The α_1C_ subunit was divided into 3 fragments based on endogenous restriction sites: KpnI in pMT2, SalI in α_1C_, SpeI in α_1C_, and a second SpeI in the 3′ untranslated region of α_1C_. The first fragment of size 2.5 kB from KpnI (pMT2)-SalI spanned the N-terminus up to domain II S6 of the channel. The last fragment of size 4 kB from SpeI-SpeI (in the 3′ untranslated region) spanned domain IIIS1 to the end of the C-terminus. Both of these fragments were separately subcloned into pBluescript SK+ (Stratagene). Specific mutagenic primers (Table S2) were designed and used in PCR-directed mutagenesis on the subcloned fragments using Stratagene's Site-directed Mutagenesis Kit. M1160L and M1161A were created using the GeneEditor Site Directed Mutagenesis Kit (Promega) with the following primers: M1160L (5′- CATTGCCTTCTTC**C**TGATGAACATC-3′) and M1161A (5′-CATTGCCTTCTTCATG**GCC**AACATCTTCGTGGG-3′). Fragments were subcloned back into the parent α_1C_ subunit in pMT2, and the mutations were confirmed by DNA sequencing.

### Cell Culture, Transfection, Electrophysiology, and Data Analysis

Cell culture and transfection using tsA201 cells are presented in the SI materials and were done as previously described [39],[40]. Whole-cell (ruptured) recordings were performed on an Axopatch 700A amplifier linked to a personal computer equipped with pClamp9 (Axon Instruments, Foster City, CA). Patch pipettes (Sutter, BF 150-86-15) were pulled using a Sutter P-87 microelectrode puller and polished with a Narashige microforge [39],[40]. Pipettes (2–4 MΩ) were filled with (in mM) 140 Cs-methanesulfonate, 4 MgCl_2_, 9 EGTA, 9 HEPES (pH 7.2 adjusted with CsOH). Bath recording solution comprised of (in mM) 20 BaCl_2_, 85 CsCl, 40 TEA-Cl, 1 MgCl_2_, 10 HEPES, 10 glucose (pH 7.3 adjusted with CsOH). Perfusions consisted of bath solution with various concentrations of DHP from 0.01 µM to 100 µM or 10 µM CdCl_2_, and applied using a gravity-driven system. In some cases, 0.1% DMSO was added to the control bath solution (no drug). Data were filtered at 1 kHz (−3 dB, 4-pole Bessel) and digitized at 2 kHz. Currents were elicited by stepping from −100 mV to the indicated test potentials. Drug-response curve were determined by depolarizing to elicit maximal inward Ba^2+^ currents (usually 0 to +10 mV) every 5 seconds, first perfusing with control solution and then with various concentrations of DHP compounds until the current amplitude reached a steady-state level. Recordings were done with Ba^2+^as Ca^2+^ may greatly affect current through mechanisms such as Ca^2+^-dependant inactivation or Ca^2+^-dependant facilitation [42]. Channel behavior was otherwise normal in the mutants as Ba^2+^ current through the mutant channels were elicited at the range of applied voltages from −30 to +40 mV, which is similar to that of the wild-type channel. Dose-response curves were fitted to the equation:
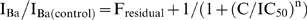
Where I_Ba_ is the current recorded either in the absence (I_Ba(control)_) or the presence of drug; F_residual_ is the fraction residual current at saturated concentrations of DHP; C is the concentration of DHP; IC_50_ is the DHP concentration required to elicit half the maximal inhibition; and n is the Hill coefficient (see Table S3 for more information). As DHPs do not physically occlude the pore [43] F_residual_ was included in the analyses so that the estimated IC_50_ accurately represents the interaction with the channel and not confounded by the efficacy of the block. Fits were performed in Origin 7 (OriginLab Corp., Northampton, MA). All experiments were performed at room temperature (∼22°C). Data are presented as mean±sem. Statistical analysis was done using one-way analysis of variance (ANOVA, Holm-Sidak *post-hoc*). Differences were considered significant if *p*<0.05.

### Chemicals and Solutions

All chemicals used in the cell culture were purchased from GIBCO (Invitrogen). Chemicals used for physiological recordings were purchased from Sigma. Nifedipine was purchased from Alamone Labs (Jerusalem, Israel) and from Sigma, and nemadipine was purchased from Chembridge Corp. (Chembridge ID#: 5619779).

## Supporting Information

Figure S1 Animals with egg laying (Egl) defects and variable abnormal (Vab) defects in response to increasing concentrations of nemadipine. (a) Dose response curves for the tr89, the domain I mutant, ad695 and n2368, the egl-19 hypermorphs, and N2, the wild-type control. (b) Dose response curves for domain II mutants. (c) Dose response curves for domain III mutants. All the mutants analyzed in the Egl and Vab assays have increased resistance to nemadipine compared to N2 showing that these mutants are DHP-resistant. The Egl and Vab assays were performed as outlined in Materials and Methods. Error bars, ±s.e.m.
(2.23 MB EPS)

Table S1 An excel file of the calculations of correlations between the worm phenotypes and the behavior of the mutant rat channels.
(0.17 MB XLS)

Table S2 Primers used in PCR-directed mutagenesis of rat alpha 1C mutants. Bold letter indicate mutations. Primers listed from 5′ to 3′.
(0.04 MB DOC)

Table S3 An excel file of the Hill coefficients of the rat channel variants in response to nifedipine.
(0.02 MB XLS)

## References

[1] Berridge MJ, Lipp P, Bootman MD (2000). The versatility and universality of calcium signalling.. Nat Rev Mol Cell Biol.

[2] Catterall WA (2000). Structure and regulation of voltage-gated Ca2+ channels.. Annu Rev Cell Dev Biol.

[3] Catterall WA, Perez-Reyes E, Snutch TP, Striessnig J (2005). International Union of Pharmacology. XLVIII. Nomenclature and structure-function relationships of voltage-gated calcium channels.. Pharmacol Rev.

[4] Sonkusare S, Palade PT, Marsh JD, Telemaque S, Pesic A (2006). Vascular calcium channels and high blood pressure: pathophysiology and therapeutic implications.. Vascul Pharmacol.

[5] Splawski I, Timothy KW, Sharpe LM, Decher N, Kumar P (2004). Ca(V)1.2 calcium channel dysfunction causes a multisystem disorder including arrhythmia and autism.. Cell.

[6] Bech-Hansen NT, Naylor MJ, Maybaum TA, Pearce WG, Koop B (1998). Loss-of-function mutations in a calcium-channel alpha1-subunit gene in Xp11.23 cause incomplete X-linked congenital stationary night blindness.. Nat Genet.

[7] Chobanian AV, Bakris GL, Black HR, Cushman WC, Green LA (2003). The Seventh Report of the Joint National Committee on Prevention, Detection, Evaluation, and Treatment of High Blood Pressure: the JNC 7 report.. Jama.

[8] Tang S, Yatani A, Bahinski A, Mori Y, Schwartz A (1993). Molecular localization of regions in the L-type calcium channel critical for dihydropyridine action.. Neuron.

[9] Grabner M, Wang Z, Hering S, Striessnig J, Glossmann H (1996). Transfer of 1,4-dihydropyridine sensitivity from L-type to class A (BI) calcium channels.. Neuron.

[10] Schuster A, Lacinova L, Klugbauer N, Ito H, Birnbaumer L (1996). The IVS6 segment of the L-type calcium channel is critical for the action of dihydropyridines and phenylalkylamines.. Embo J.

[11] Peterson BZ, Johnson BD, Hockerman GH, Acheson M, Scheuer T (1997). Analysis of the dihydropyridine receptor site of L-type calcium channels by alanine-scanning mutagenesis.. J Biol Chem.

[12] Sinnegger MJ, Wang Z, Grabner M, Hering S, Striessnig J (1997). Nine L-type amino acid residues confer full 1,4-dihydropyridine sensitivity to the neuronal calcium channel alpha1A subunit. Role of L-type Met1188.. J Biol Chem.

[13] Ito H, Klugbauer N, Hofmann F (1997). Transfer of the high affinity dihydropyridine sensitivity from L-type To non-L-type calcium channel.. Mol Pharmacol.

[14] Hockerman GH, Peterson BZ, Johnson BD, Catterall WA (1997). Molecular determinants of drug binding and action on L-type calcium channels.. Annu Rev Pharmacol Toxicol.

[15] Lacinova L, Klugbauer N, Hu M, Hofmann F (1999). Reconstruction of the dihydropyridine site in a non-L-type calcium channel: the role of the IS6 segment.. FEBS Lett.

[16] Peterson BZ, Tanada TN, Catterall WA (1996). Molecular determinants of high affinity dihydropyridine binding in L-type calcium channels.. J Biol Chem.

[17] Mitterdorfer J, Wang Z, Sinnegger MJ, Hering S, Striessnig J (1996). Two amino acid residues in the IIIS5 segment of L-type calcium channels differentially contribute to 1,4-dihydropyridine sensitivity.. J Biol Chem.

[18] Wappl E, Mitterdorfer J, Glossmann H, Striessnig J (2001). Mechanism of dihydropyridine interaction with critical binding residues of L-type Ca2+ channel alpha 1 subunits.. J Biol Chem.

[19] Berjukow S, Marksteiner R, Gapp F, Sinnegger MJ, Hering S (2000). Molecular mechanism of calcium channel block by isradipine. Role of a drug-induced inactivated channel conformation.. J Biol Chem.

[20] Glossmann H, Ferry DR, Goll A, Striessnig J, Zernig G (1985). Calcium channels and calcium channel drugs: recent biochemical and biophysical findings.. Arzneimittelforschung.

[21] Peterson BZ, Catterall WA (2006). Allosteric interactions required for high-affinity binding of dihydropyridine antagonists to Ca(V)1.1 Channels are modulated by calcium in the pore.. Mol Pharmacol.

[22] Wang X, Du L, Peterson BZ (2007). Calcicludine binding to the outer pore of L-type calcium channels is allosterically coupled to dihydropyridine binding.. Biochemistry.

[23] Mitterdorfer J, Sinnegger MJ, Grabner M, Striessnig J, Glossmann H (1995). Coordination of Ca2+ by the pore region glutamates is essential for high-affinity dihydropyridine binding to the cardiac Ca2+ channel alpha 1 subunit.. Biochemistry.

[24] Peterson BZ, Catterall WA (1995). Calcium binding in the pore of L-type calcium channels modulates high affinity dihydropyridine binding.. J Biol Chem.

[25] Yamaguchi S, Okamura Y, Nagao T, Adachi-Akahane S (2000). Serine residue in the IIIS5–S6 linker of the L-type Ca2+ channel alpha 1C subunit is the critical determinant of the action of dihydropyridine Ca2+ channel agonists.. J Biol Chem.

[26] Yamaguchi S, Zhorov BS, Yoshioka K, Nagao T, Ichijo H (2003). Key roles of Phe1112 and Ser1115 in the pore-forming IIIS5–S6 linker of L-type Ca2+ channel alpha1C subunit (CaV 1.2) in binding of dihydropyridines and action of Ca2+ channel agonists.. Mol Pharmacol.

[27] Kwok TC, Ricker N, Fraser R, Chan AW, Burns A (2006). A small-molecule screen in C. elegans yields a new calcium channel antagonist.. Nature.

[28] Burns AR, Kwok TC, Howard A, Houston E, Johanson K (2006). High-throughput screening of small molecules for bioactivity and target identification in Caenorhabditis elegans.. Nat Protoc.

[29] Lee RY, Lobel L, Hengartner M, Horvitz HR, Avery L (1997). Mutations in the alpha1 subunit of an L-type voltage-activated Ca2+ channel cause myotonia in Caenorhabditis elegans.. Embo J.

[30] Mathews EA, Garcia E, Santi CM, Mullen GP, Thacker C (2003). Critical residues of the Caenorhabditis elegans unc-2 voltage-gated calcium channel that affect behavioral and physiological properties.. J Neurosci.

[31] Bean BP (1984). Nitrendipine block of cardiac calcium channels: high-affinity binding to the inactivated state.. Proc Natl Acad Sci U S A.

[32] Soldatov NM, Zhenochin S, AlBanna B, Abernethy DR, Morad M (2000). New molecular determinant for inactivation of the human L-type alpha1C Ca2+ channel.. J Membr Biol.

[33] Shi C, Soldatov NM (2002). Molecular determinants of voltage-dependent slow inactivation of the Ca2+ channel.. J Biol Chem.

[34] Hohaus A, Beyl S, Kudrnac M, Berjukow S, Timin EN (2005). Structural determinants of L-type channel activation in segment IIS6 revealed by a retinal disorder.. J Biol Chem.

[35] Splawski I, Timothy KW, Decher N, Kumar P, Sachse FB (2005). Severe arrhythmia disorder caused by cardiac L-type calcium channel mutations.. Proc Natl Acad Sci U S A.

[36] Waggoner LE, Zhou GT, Schafer RW, Schafer WR (1998). Control of alternative behavioral states by serotonin in Caenorhabditis elegans.. Neuron.

[37] Sulston JE, Albertson DG, Thomson JN (1980). The Caenorhabditis elegans male: postembryonic development of nongonadal structures.. Dev Biol.

[38] Garcia LR, Mehta P, Sternberg PW (2001). Regulation of distinct muscle behaviors controls the C. elegans male's copulatory spicules during mating.. Cell.

[39] Feng ZP, Hamid J, Doering C, Bosey GM, Snutch TP (2001). Residue Gly1326 of the N-type calcium channel alpha 1B subunit controls reversibility of omega-conotoxin GVIA and MVIIA block.. J Biol Chem.

[40] Hui K, Gardzinski P, Sun HS, Backx PH, Feng ZP (2005). Permeable ions differentially affect gating kinetics and unitary conductance of L-type calcium channels.. Biochem Biophys Res Commun.

[41] Lansman JB, Hess P, Tsien RW (1986). Blockade of current through single calcium channels by Cd2+, Mg2+, and Ca2+. Voltage and concentration dependence of calcium entry into the pore.. J Gen Physiol.

[42] Halling DB, Aracena-Parks P, Hamilton SL (2006). Regulation of voltage-gated Ca2+ channels by calmodulin.. Sci STKE.

[43] Hockerman GH, Johnson BD, Scheuer T, Catterall WA (1995). Molecular determinants of high affinity phenylalkylamine block of L-type calcium channels.. J Biol Chem.

