# Correction: A Genetic Screen for Dihydropyridine (DHP)-Resistant Worms Reveals New Residues Required for DHP-Blockage of Mammalian Calcium Channels

**DOI:** 10.1371/annotation/c38f7645-c9d8-4371-b363-5a9099c75e83

**Published:** 2009-02-24

**Authors:** Trevor C. Y. Kwok, Kwokyin Hui, Wojciech Kostelecki, Nicole Ricker, Guillermo Selman, Zhong-Ping Feng, Peter John Roy

There were errors in the Author Contributions. The correct contributions are: Conceived and designed the experiments: TK KH ZPF PR. Performed the experiments: TK KH WK NR GS. Analyzed the data: TK KH ZPF PR. Wrote the paper: TK KH ZPF PR.

