# Supplementary material for: A Genetic Screen for Dihydropyridine (DHP)-Resistant Worms Reveals New Residues Required for DHP-Blockage of Mammalian Calcium Channels
Source: PLoS Genet. 2008 May 9;4(5):e1000067. doi: 10.1371/journal.pgen.1000067 (PMC2362100; doi:10.1371/journal.pgen.1000067)
Supplement: Table S2 — Primers used in PCR-directed mutagenesis of rat alpha 1C mutants. Bold letter indicate mutations. Primers listed from 5′ to 3′. (0.04 MB DOC) [file pgen.1000067.s003.doc]

**Supplemental Table 2.** Primers used in PCR-directed mutagenesis of rat α_1C_ mutants. Bold letter indicate mutations. Primers listed from 5’ to 3’.
